# Theories and Practice

**Published:** 1920-05-01

**Authors:** 


### THE GRAFTING OF BONE AND CARTILAGE.

### Theories and Practice.

Medical and surgical advances for which the war was directly responsible have been so numerous that it seems impossible that the direct effect will appreciably decline for many years, and an excellent illustration of this continued influence is seen in the spheres of bone surgery. For months after hostilities ceased, cases of ununited fractures, etc., have come up for surgical consideration, the delay being due to the fact that a large number were not at first in a fit state to be operated upon, and that a relative asepsis of the patients' lesions was a preliminary necessity. The frequent success and the obvious value of bone grafting have long been recognised, but upon the questions of exact technique, the method of bone regeneration, the portions of a graft which may survive are matters upon which there has been remarkably little general agreement.

### Recent Investigations.

One of the most extensive observational papers published at the later stages of the war was that of Berg and Thalhimer in 1918, who were directly opposed to Macewen's teaching that periosteum was merely a limiting membrane without bone-forming capacity, and they again emphasised the fact that all cartilage bone is produced by cells arising from the osteoblasts lining the periosteum and is deposited in preformed cartilage, the latter being absorbed. Without going deeply into' their histological findings we may summarise their results as follows. Periosteum devoid of adherent bone cells produces bones when transplanted even into foreign tissues, and endosteum and osteoblasts lining the Haversian systems in the grafted pieces produce bone very actively. Transplanted bone is absorbed not only by osteoblasts but also by the direct action of growing young bone, and the transplanted bone is replaced either by a gradual advance of the new bone or by a separate extension of the new bone into the substance of the graft. Also marrow and marrow spaces develop in the bone which develops from the transplanted periosteum.

This finding has been partially confirmed by animal feeding experiments; but, whatever the exact process by which the bone graft typically acts, it seems that the periosteum is not absolutely necessary in grafting, whether it be an osteogenetic layer or not. In a number of cases the effect of previously boiling the autoplastic graft has been tried, and so successful have the results been that it seems only fair to assume that such graft must be replaced by invasion and vascularisation from the adjacent bone. Certainly there is the advantage that this type of prepared graft can be prepared more accurately and cut to any size and shape, and a certain amount of use has been made of the method in connection with reconstructional surgery of the jaws and face, but we believe we are right in claiming that a higher percentage success has been obtained with fresh autoplastic grafts.

One curious explanation has been put forward, namely, that osseous regeneration in adults is essentially a pathological phenomenon, inflammatory in nature, and therefore absolutely different from the original growth of the bones. In a large number of war cases there has undoubtedly been some degree of latent infection, and the suggestion that the inflammation produced, when mild, may be a help towards more profuse bone production is particularly interesting. The originators of this idea quote Tixier's method of treating pseudo-arthrosis by introducing a mild infection so as to stimulate osteogenesis, and add that the use of plates in the early stages of compound fractures would be justifiable on the same grounds. In any case, it has been a common experience that some of the best results of grafting have paradoxically occurred, in spite of— or because of, a mild degree of septic irritation, carefully kept under restraint. Upon such a point, however, we reserve our judgment until more work on the new lines has been done, until civilian hospital conditions and a true comparison between the results of this and the rigid aseptic teaching can be drawn.

### The End Results.

Whatever method of fixing the graft be used, whether it be let into grooves cut in the adjacent bones or whether the bone ends are trimmed and fixed into the graft, whether wires, etc., are used or not, it seems probable that the real end result of a graft cannot be definitely determined until years after the actual operation. Many consider that the introduction of any wires or deep fixation sutures is absolutely contra-indicated, and that the best results are obtained when the graft is simply wedged into place and the position of the affected part maintained by rigid splinting. On the idea which was urged by Imbert in 1918, that in any fracture or pseudo-arthrosis, the latent power of osteogenesis undoubtedly exists, and that it is the *stimulus* rather than the rigidity of the graft which produces vigorous and firm union, the omission of all accessory wiring, etc., would be the rational procedure, and the importance attached to perfect splinting and extreme extension of relatively paramount importance. One interesting point is made by some French observers, who have watched the healing process by means of periodic radiographs, that some grafts actually live and persist as part of the new bony structure, not being replaced by ingrowth from without. The particular grafts were completely denuded of periosteum, and in two notable cases a later fracture of the graft occurred—but regeneration of bone from the actual graft ends occurred as the second healing process continued. This has not, however, been the general experience, and on the width of the interfragmentary space depends the advisability of using a graft, as such fractures have generally meant failure more or less complete.

**The Grafting of Bone and Cartilage**—(*continued*).

CARTILAGE GRAFTING.

Generally speaking, there is reasonable agreement that success is only obtained when fibro-cartilaginous mainly or fibrous grafts are employed, and that, in any case, it is only this portion of the structure which survives. Otherwise the pieces are rapidly attacked by leucocytes, and all that ultimately remains is a fibrous mass. Conspicuously has this resulted in the case of those skull injuries and cranial defects, after gunshot wounds, closures of which have been attempted with pieces of cartilage. Sometimes bony closure has been seen, but it would appear that the graft acted merely in a stimulating capacity. Much better results were seen with the tibial osteoperiosteal type, *i.e.*, scales raised from the tibial surface, so that bony spicules remain attached to the membrane. With this technique nearly 100 per cent. success has been claimed, and undoubtedly this type of light grafting is greatly superior to the use of true cartilage.

OTHER CONCLUSIONS.

Among other findings in connection with this branch of reconstructional surgery the conclusions published by Wheeler in the *British Medical Journal* 1919, i. 119, are worthy of attention, and from among them the following may be abstracted.

In the first place the detailed effect of the graft is directly influenced by the strains and stresses to which it is subjected, and the amount of growth in the bone depends on the need for it. The periosteum should be left on the graft, because, although not essential, it is the medium through which new blood-vessels enter the graft and surrounding structures. Furthermore, in removing the periosteum, superficial layers of osteoblasts may be sacrificed. A periosteal-covered graft is less likely to become rapidly absorbed.

After three months' fixation, it is advisable to commence first passive then mild active movements, but remember both at this stage and during operation that brittle or sclerosed bone makes an unsuitable soil for implanting the graft, and firm union cannot be expected with it; in such cases a periosteal-covered graft, instead of exhibiting osteogenetic power and responding to Wolff's law of strains and the growth of bones, may become attenuated and absorbed or break in the critical area five or six months after operation.

The method of using an intermedullary peg is apparently only of value in the case of fractures of the radius and ulnar. In the case of the humerus and femur, long stout inlay grafts give best results. Sliding grafts should only be employed in simple, fresh cases. Absolute fixation of the graft in its bed, either as part of the operation, or afterwards by splints or plaster, is essential to success. The greatest example of uniformly good results is seen in grafting for spinal caries, but here the spinous processes, into which the graft is inserted, are *healthy*, normal bone, and herein probably lies the secret of most of the success that we can justifiably expect to arise from bone grafting as applied to orthopædic surgery.

